# Absence of xenotropic murine leukaemia virus-related virus in Danish patients with multiple sclerosis

**DOI:** 10.1186/1742-4690-8-S1-A213

**Published:** 2011-06-06

**Authors:** Romana Maric, Finn S Pedersen, Anders Kjeldbjerg, Anné Moeller-Larsen, Shervin Bahrami, Tomasz Brudek, Thor Petersen, Tove Christensen

**Affiliations:** 1 Department of Medical Microbiology and Immunology, Aarhus University, Aarhus, DK-8000 C, Denmark; 2 Department of Molecular Biology, Aarhus University, Aarhus, DK-8000 C, Denmark; 3 Department of Neurology, Aarhus University Hospital, Aarhus, DK-8000 C, Denmark

## Background

Detection of a novel Gammaretrovirus, Xenotropic Murine leukaemia virus-Related Virus (XMRV) has been reported in peripheral blood mononuclear cells of patients with chronic fatigue syndrome, and in prostate cancer tissue. As Multiple Sclerosis (MS) is a disease with retroviral association (human endogenous retroviruses (HERVs)), we investigated whether XMRV could be contributing to MS aetiology by testing a well defined cohort of Danish MS patients for the presence of XMRV sequences.

## Materials and methods

We have analysed DNA samples isolated from peripheral blood mononuclear cells (PBMCs) from 50 Danish patients with clinically well-characterized MS for evidence of the presence of XMRV gag or env sequences by PCR, using concomitant amplification of the cellular GAPDH gene as control.

## Results

In this study, which included relevant positive and negative isolation controls and PCR controls, we failed to detect XMRV sequences in PBMCs from Danish MS patients.

## Conclusions

There is no apparent association between XMRV infection and MS in Denmark.

